# NK and NKT Cell Depletion Alters the Outcome of Experimental Pneumococcal Pneumonia: Relationship with Regulation of Interferon-*γ* Production

**DOI:** 10.1155/2015/532717

**Published:** 2015-05-31

**Authors:** Eirini Christaki, Evdoxia Diza, Evangelos J. Giamarellos-Bourboulis, Nikoletta Papadopoulou, Aikaterini Pistiki, Dionysia-Irini Droggiti, Marianna Georgitsi, Alzbeta Machova, Dimitra Lambrelli, Nicolaos Malisiovas, Pavlos Nikolaidis, Steven M. Opal

**Affiliations:** ^1^First Department of Medicine, AHEPA University Hospital, Thessaloniki, Greece; ^2^Infectious Diseases Division, Alpert Medical School of Brown University, Providence, RI, USA; ^3^Department of Microbiology, Aristotle University of Thessaloniki Medical School, Thessaloniki, Greece; ^4^4th Department of Internal Medicine, Medical School, University of Athens, Athens, Greece; ^5^Institute for Medical Microbiology, Immunology and Hygiene, University of Cologne, Cologne, Germany; ^6^Department of Economics, University of Macedonia, Thessaloniki, Greece

## Abstract

*Background*. Natural killer (NK) and natural killer T (NKT) cells contribute to the innate host defense but their role in bacterial sepsis remains controversial. *Methods*. C57BL/6 mice were infected intratracheally with 5 × 10^5^ cfu of *Streptococcus pneumoniae*. Animals were divided into sham group (Sham); pretreated with isotype control antibody (CON) group; pretreated with anti-asialo GM1 antibody (NKd) group; and pretreated with anti-CD1d monoclonal antibody (NKTd) group before bacterial challenge. Serum and tissue samples were analyzed for bacterial load, cytokine levels, splenocyte apoptosis rates, and cell characteristics by flow cytometry. Splenocyte miRNA expression was also analyzed and survival was assessed. *Results*. NK cell depletion prolonged survival. Upon inhibition of NKT cell activation, spleen NK (CD3−/NK1.1+) cells increased compared to all other groups. Inhibition of NKT cell activation led to higher bacterial loads and increased levels of serum and splenocyte IFN-*γ*. Splenocyte miRNA analysis showed that miR-200c and miR-29a were downregulated, while miR-125a-5p was upregulated, in anti-CD1d treated animals. These changes were moderate after NK cell depletion. *Conclusions*. NK cells appear to contribute to mortality in pneumococcal pneumonia. Inhibition of NKT cell activation resulted in an increase in spleen NK (CD3−/NK1.1+) cells and a higher IFN-*γ* production, while altering splenocyte miRNA expression.

## 1. Introduction

Severe sepsis and septic shock are a leading cause of death worldwide. Community-acquired pneumonia (CAP) due to* Streptococcus pneumoniae* accounts for a large number of cases [1, 2]. Current theories regarding pathogenesis suggest that sepsis develops as an overwhelming response of the host innate immune system to a microbial stimulus. However, most of research has been focused on the role of blood monocytes and tissue macrophages as effectors of this response whereas little information has been generated for the role of natural killer (NK) cells. NK cells are effector cells of the innate immune response and they secrete both proinflammatory cytokines like tumor necrosis factor-alpha (TNF-*α*) and interferon-gamma (IFN-*γ*) and anti-inflammatory cytokines like transforming growth factor-*β* (TGF-*β*) and IL-10 depending on the eliciting stimulus [3]. As such, NK cells regulate host responses by driving cytokine release and leading to T cell priming towards a Th1 or Th2 immune response, whereas they are also involved in the initiation of apoptosis [4].

Like NK cells, NKT cells are identified as regulators of the innate immune response. NKT cells recognize glycolipid antigens presented by the CD1d molecule [5]. NKT cells express NK-1.1, DX5, Ly49A markers, and components of the T cell receptor (TCR) and share characteristics of NK and T cells. iNKT cells are a subset of NKT cells that express one TCR that consists of a restricted *β* chain and an invariant V*α*14/Ja18 chain in mice or a Va24/Ja18 in humans [6, 7]. A recent study showed that, after* S. pneumoniae* infection, cytokine production by V*α*14*i*NKT cells was dependent on antigen presentation by the CD1d molecule. The investigators described a critical element of antigen recognition by the conserved TCR of iNKT cells which was the requirement of a hexose sugar *α*-linked to a microbial lipid, serving as a microbe associated molecular pattern [8]. NKT cells can also elicit a rapid IFN-*γ* release after bacterial infection [5, 8, 9]. These experimental data indicate that the NK/NKT cell balance may play a major role in the pathogenesis of CAP by* S. pneumoniae*.

Little is known about the involvement of specific miRNAs in the regulation of the innate immune response. Global miRNA expression profiling revealed that miR-150 and miR-155 were specifically expressed in lymphoid lineage cells, including NK cells [10, 11]; in addition, miR-150 was found to contribute to iNKT cell development [12] and miR-155 to NK cell IFN-*γ* production [13]. To this end, we investigated the role of NK and NKT cells in an experimental murine pneumococcal pneumonia model of sepsis. We examined the effect of NK cell depletion and inhibition of NKT cell activation on cytokine stimulation and on specific microRNA response in pneumococcal sepsis. Our hypothesis was that since NK and NKT cells play an immunoregulatory role in sepsis,* in vivo* depletion of these cell populations could affect mortality.

## 2. Materials and Methods

### 2.1. Animals

Experiments were carried out in eight to twelve weeks old, 25 gr body weight, specific-pathogen-free C57BL/6 wild-type mice (Institute Pasteur, Athens, Greece) using the standard pneumococcal pneumonia model of experimental sepsis [14]. Experiments were performed in the Laboratory for Experimental Medicine of Attikon University General Hospital. After acclimatization, mice were kept in cages with constant rotation rate of 70 air-changes per hour to ensure sterility. Mice were fed standard chow (type 4rf 18) and were allowed water* ad libitum*. They were kept in an isolated room with a controlled temperature of 24°C and a day–night cycle of 8 am–8 pm. The study was approved by the Veterinary Directorate of the Prefecture of Athens, Greece (approval *Κ*/7570).

### 2.2. Experimental Design

The animals were experimentally infected with an invasive, smooth, encapsulated, highly susceptible strain of* Streptococcus pneumoniae* (clinical specimen isolated from blood). The bacterial suspension was grown logarithmically overnight at 37°C in trypticase soy broth, washed, and resuspended in phosphate buffered saline (PBS) (Merck, Darmstadt, Germany). Based on preliminary experiments, the lethal dose 75 (LD_75_) was 5 × 10^5^ cfu/mouse; this was used for further experiments. Mice were lightly anaesthetized with diethyl ether (Alter Chem, Athens, Greece), suspended at a 60° angle using their front incisors; a volume of 50 *μ*L* S. pneumoniae* suspension was instilled under direct visualization into the glottis, and it was aspirated into the lower respiratory tract.

Animals were randomly assigned into four groups:

- Group Sham, sham-operated mice that received intratracheal installation of normal saline.
- Group CON, control mice; these mice were intravenously pretreated 24 hours prior to bacterial challenge with 50 *μ*L (1 mg/kg) of nonspecific IgG2b, *κ* isotype control antibody (BD Pharmingen, San Diego, CA).
- Group NKd, NK-depleted mice; these mice were iv pretreated 24 hours prior to bacterial challenge with 50 *μ*L of anti-asialo GM1 rabbit polyclonal antibody (Wako Chemicals GmbH, Neuss, Germany) in order to achieve NK cell inactivation. This anti-asialo GM1 has been shown to effectively deplete NK cells in mice for 4 days so one additional injection of the same dose was given on day 5 of the experiment [15, 16].
- Group NKTd, NKT-depleted mice; these mice were intravenously pretreated 24 hours prior to bacterial challenge with 50 *μ*L (2 mg/kg) of the monoclonal antibody anti-CD1d, clone 1B1 (BD Pharmingen, San Diego, CA) [17].

On each day of the experiment, 2 to 3 mice per group were studied. Half animals per group were monitored for 7 days for signs of sepsis (lethargy, apathy, loss of mobility, piloerection, diarrhea, tachypnea, periorbital exudates, etc.) and deaths were recorded.

In a separate set of experiments, after* in vivo* NK and NKT cell depletion, all animals were euthanized 48 hours after bacterial inoculation, a point at which animals are expected to have developed sepsis due to pneumococcal pneumonia. Sacrifice was done by inhalation of diethyl ether followed by ketamine intramuscular injection. At sacrifice, one midline abdominal incision was performed and blood was sampled from the lower vena cava under aseptic conditions. Blood was placed into sterile and EDTA-coated tubes (Vacutainer, BD, Cockeysville, MD). Specimens of liver, spleen, and right lung were excised and put into separate sterile containers.

### 2.3. Splenocyte Preparation and Cell Surface Phenotype Analysis

Spleens were carefully dissected from each animal, kept in 1 mL RPMI 1640 (Biochrom, Berlin, Germany) at 0°C, immediately homogenized, filtered (250 *μ*m Nylon Sieve, Alter Chem, Athens, Greece), centrifuged at 1700 rpm for 5 minutes, and resuspended in 1 mL RPMI 1640. Cells were counted using trypan blue and a Neubauer hematocytometer (Poly Optik GmbH, Germany). Splenocytes were incubated in the dark for 15 min with the monoclonal antibodies anti-mouse-CD3 (clone 145-2C11) at the fluorochrome fluorescein isothiocyanate (FITC, emission 525 nm; Cell Lab, Beckman Coulter Inc., Miami, FL, USA); with the monoclonal antibody anti-mouse NK1.1 (clone: PK136) at the fluorochrome phycoerythrin (PE, emission 575 nm; Cell Lab, Beckman Coulter Inc., Miami, FL, USA); and with anti-mouse-CD1d (clone: 1B1) (PE, emission 575 nm; Biolegend, San Diego, CA, USA). Cells were lysed with VersaLyse Solution (Beckman Coulter Inc., Miami, FL, USA), washed with PBS (Merck, Darmstadt, Germany), centrifuged, and resuspended at PBS with 0.16% formaldehyde. Cells were analyzed by flow cytometry using an FC-500 instrument (Beckman Coulter Inc., Miami, FL), with gating for lymphocytes based on their characteristic FS/SS scattering. Cells staining negative for CD3 (−) and positive for N*Κ*1.1 (+) were considered N*Κ* cells.

### 2.4. Apoptosis

The rate of apoptosis of spleen lymphocytes and macrophages was determined after cell staining for the protein Annexin-V at the fluorochrome FITC (emission 525 nm; Cell Lab, Beckman Coulter Inc., Miami, FL, USA) and for propidium iodide (PI) at the fluorochrome Texas Red ECD (emission 613 nm, Invitrogen, OR, USA) followed by flow cytometric analysis. Cells were analyzed at the FC-500 (Beckman Coulter Co., FL, USA), after separate gating for lymphocytes and for macrophages by their characteristic forward and side scattering. Cells staining positive for Annexin-V (+) and negative for PI (−) were considered apoptotic.

### 2.5. *Ex Vivo* Splenocyte Stimulation

Splenocytes (5 × 10^6^ cells/well) suspended in growth medium (RPMI with 100 U/mL penicillin and 0.1 mg/mL streptomycin, Sigma Co., St Louis, MO, USA) were incubated at 37°C, 5% CO_2_ in the presence or absence of 100 pg/mL of IL-2 (R&D Systems Inc., Minneapolis, MN, USA); 100 pg/mL of IL-12 (R&D Systems Inc.), or 10 ng/mL of lipopolysaccharide (LPS) of* Escherichia coli* O55:B5 (Sigma Co., St Louis, MO, USA) for 24 or 48 hours. At those time-points, plates were centrifuged at 1300 rpm for 7 min and supernatants were collected and kept at −80°C until cytokine analysis was performed.

### 2.6. Cytokine Analysis

After* ex vivo* stimulation, 24-hour splenocyte supernatants were tested for TNF-*α* with ELISA DuoSet mouse TNF-*α* (Janssen R&D, NJ, USA) and 48-hour supernatants for IFN-*γ* and IL-10 with Mouse IFNg “Femto-HS” High Sensitivity ELISA Ready-Set-Go (eBioscience, Ltd., San Diego, CA, USA) and mouse IL-10 ELISA Ready-Set-Go! (eBioscience, LtD, San Diego, CA, USA), respectively. IFN-*γ* was also measured in serum samples. The lower limits of detection were 62.5 pg/mL for TNF-*α*; 30 pg/mL for IL-10; and 0.75 pg/mL for IFN-*γ*.

### 2.7. Total RNA Isolation

Total RNA from mouse splenocytes was isolated using TRIzol according to the manufacturer's protocol (Invitrogen, Life Technologies, Grand Island, NY, USA). Quantity and quality of total RNA were determined by UV spectrophotometric reading on the nanovue (GE Healthcare, Waukesha, WI, USA) and automated gel electrophoresis on the Experion system (Bio-Rad, Hercules, CA, USA), respectively. Samples with RNA quality indicator (RQI) more than 8.0 were used for gene expression TaqMan assays.

### 2.8. TaqMan miRNA and mRNA Assays

Mature miRNA expression was quantified using TaqMan microRNA assays (ABI, Life Technologies, Carlsbad, CA, USA). Total RNA (5 ng) was reverse-transcribed using miRNA specific primers and the TaqMan Reverse Transcription Kit (ABI, Life Technologies, Carlsbad, CA, USA). TaqMan miRNA assays were performed on a LC480 LightCycler (Roche, Basel, Switzerland), using the TaqMan Universal PCR Master Mix (*ΑΒ*Ι, Life Technologies, Carlsbad, CA, USA), and analyzed with the LC480 analysis software. Values were normalized to the endogenous control sno202. For quantification of IFN-*γ* mRNA expression, 10 ng RNA was transcribed using iScript cDNA synthesis kit (Bio-Rad, Hercules, CA, USA) according to the manufacturer's instructions. Expression of IFN-*γ* was analyzed using TaqMan gene expression assays and the data was normalized to GAPDH endogenous control. Relative fold changes of miRNA or mRNA expression were determined with the 2^−ΔΔCT^ method [18]. Mmu-miRNA and mRNA detection assays were obtained from Applied Biosystems: sno202 (assay ID: TM 001232); miR-125a-5p (assay ID: TM 002198); miR-155 (assay ID: TM 002571); miR-200c (assay ID: TM 002300); miR-29a (assay ID: TM002112); IFN-*γ* mRNA (assay ID: Mm 01168134_m1); and GAPDH (assay ID: Mm 99999915_g1).

### 2.9. Quantitative Bacterial Cultures

Specimens from lung (right lower lobe) and liver were separately weighted, homogenized, and serially diluted (1 : 10) five times. A volume of 100 *μ*L of each dilution was plated onto blood agar and incubated at 37°C, 5% CO_2_ for 24 hours before colonies were counted. Results were expressed as the log⁡10 value of colony forming units per gram of tissue (cfu/g).

### 2.10. Statistics

Data was expressed as mean ± standard error (SE). Survival statistics were analyzed and plotted with the Kaplan-Meier method and differences in survival between groups were compared by the log-rank test. Comparisons of numeric data between groups were made using the Kruskal-Wallis test. A *p* value less than 0.05 after adjustment for multiple comparisons according to Bonferroni was considered significant.

## 3. Results

**Figure 1:**
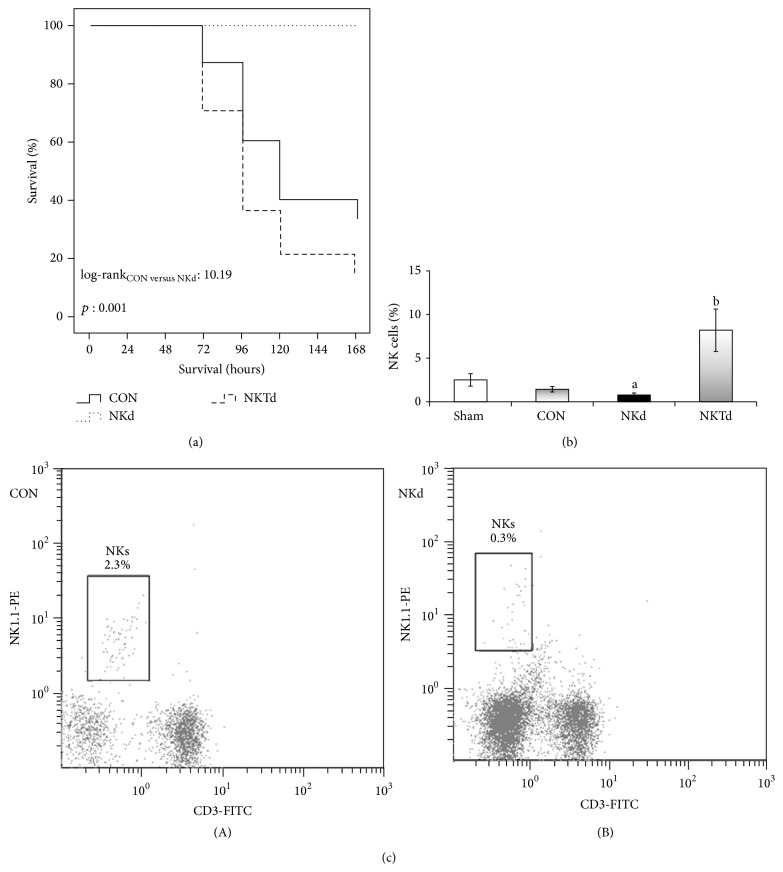
Kaplan-Meier survival plots. (a) Mice were intratracheally challenged with* Streptococcus pneumoniae* after pretreatment with nonspecific IgG (CON, *n* = 15); after pretreatment with anti-asialo GM1 antibody (NKd, *n* = 10); or after pretreatment with anti-CD1d antibody (NKTd, *n* = 14). Survival was recorded for 7 days after bacterial challenge. Ten sham-operated mice (Sham) were also followed up and none died. Comparisons log-rank_CON  VS  NKd_: 10.19, *p*: 0.001; log-rank_CON  VS  NKTd_: 1.96, *p*: 0.161; and log-rank_NKd  VS  NKTd_: 16.62; *p* < 0.0001. (b) CD3−/NK1.1+ cell populations among splenocytes from each group. Results come from *n* = 5 per group at the time of sacrifice, 48 hours after bacterial challenge. *p* of comparisons with sham-operated mice: ^a^0.018; ^b^0.030. (c) Data represent fluorescence-activated cell sorting plots obtained 48 hours after bacterial inoculation. Splenocytes were stained with fluorochrome labeled anti-NK1.1 and anti-CD3 monoclonal antibodies. CD3−/NK1.1+ (NK) cell populations in (A) control and (B) anti-asialo GM1 treated animals are depicted.

All animals of the group NKd that received the anti-asialo GM1 antibody survived. This group showed improved survival when compared to groups CON and NKTd (*p*: 0.001 and *p* < 0.001, resp.). The mortality rate of the control group was in accordance with the one expected in this experimental model of sepsis [14, 19]. All the animals that received sham intratracheal installation of normal saline survived to the end of the study period (Figure 1(a)). In mice of the NKTd group, there was a significant increase in spleen NK (CD3−/NK1.1+) cells (Figure 1(b)). Flow cytometry was used to verify NK cell depletion by anti-asialo GM1 antibody (Figure 1(c)).

**Figure 2:**
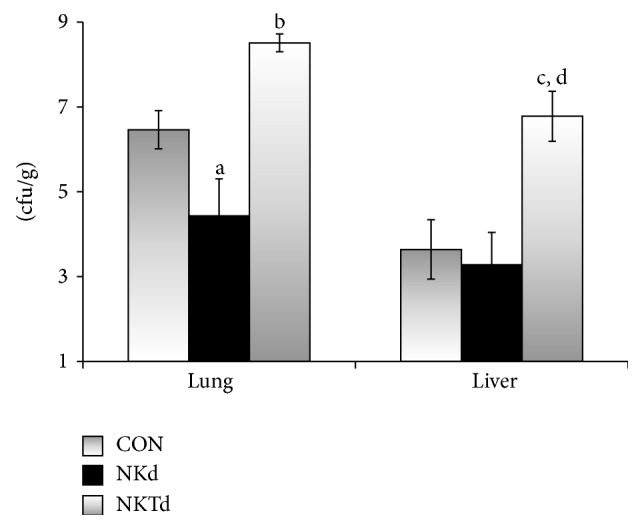
Tissue outgrowth of* Streptococcus pneumonia*. Bacterial growth was measured in the lower lobe of the right lung and in the liver after animal sacrifice 48 hours after bacterial challenge with* Streptococcus pneumoniae*. Mice were pretreated with nonspecific IgG (CON, *n* = 6); with anti-asialo GM1 antibody (NKd, *n* = 8); or with anti-CD1d antibody (NKTd, *n* = 10). Statistically significant comparisons after corrections for multiple tests are indicated: ^a^ *p*_NKd  VS  CON_: 0.044; ^b^ *p*_NKTd  VS  NKd_: 0.033; ^c^ *p*_NKTd  VS  CON_: 0.037; ^d^ *p*_NKTd  VS  NKd_: 0.012.

**Figure 3:**
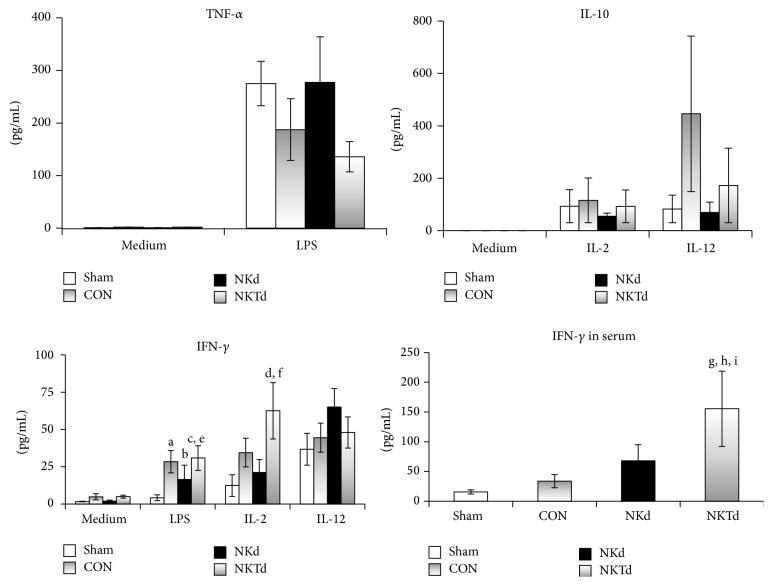
Cytokine production from splenocytes. Splenocytes were isolated after animal sacrifice 48 hours after bacterial challenge with* Streptococcus pneumoniae*. Mice were pretreated with nonspecific IgG (CON, *n* = 13); with anti-asialo GM1 antibody (NKd, *n* = 12); or with anti-CD1d antibody (NKTd, *n* = 10). Results are compared with sham-operated mice (Sham, *n* = 10). Concentrations of tumour necrosis factor-alpha (TNF-*α*), of interleukin (IL)-10 and of interferon-gamma (IFN-*γ*) were measured in supernatants after stimulation with lipopolysaccharide (LPS) of* Escherichia coli* O55:B5, with IL-2 and with IL-12. Serum concentrations of IFN-*γ* at the time of animal sacrifice are also shown. Statistically significant comparisons after corrections for multiple tests are indicated: ^a^ *p*_Sham  VS  CON_: 0.010; ^b^ *p*_Sham  VS  NKd_: 0.033; ^c^ *p*_Sham  VS  NKTd_: 0.013; ^d^ *p*_Sham  VS  NKTd_: 0.049; ^e^ *p*_NKd  VS  NKTd_: 0.049; ^f^ *p*_NKd  VS  NKTd_: 0.037; ^g^ *p*_Sham  VS  NKTd_: 0.002; ^h^ *p*_CON  VS  NKTd_: 0.009; ^i^ *p*_NKd  VS  NKTd_: 0.039.

Prolonged survival after depletion of NK cells was related to reduced outgrowth of* S. pneumoniae* in the lung; the opposite was observed in animals where CD1d activation of NKT cells was blocked (Figure 2). In order to investigate a mechanistic explanation behind the survival benefit in the animals depleted of NK cells and disadvantage in those deprived of CD1d-dependent NKT cell activation, we hypothesized that an imbalance of NK/NKT cells may affect the local and systemic dissemination of the inoculated pathogen. A change in NK and NKT cell populations could potentially influence the apoptosis of tissue macrophages or it could affect the type of cytokine profile that determines the host innate immune response to* S. pneumoniae*. Depletion of NK cells and blockade of CD1d-dependent NKT cell activation did not change the rate of spleen lymphocyte and macrophage apoptosis (data not shown). Also, there were no significant differences between the groups in regard to TNF-*α* and IL-10 production by splenocytes after stimulation with LPS, IL-2, and IL-12, although there was a trend for decreased IL-10 production after stimulation with IL-12 in the anti-asialo GM1 treated group compared with controls. However, when splenocytes were stimulated* ex vivo*, production of IFN-*γ* differed between the animal groups. More precisely, in the event of inhibition of NKT cell activation, that is, of predominance of NK cells, an exaggerated production of IFN-*γ* was found (Figure 3). This was also reflected in the concentrations of circulating IFN-*γ* (Figure 3).

**Figure 4:**
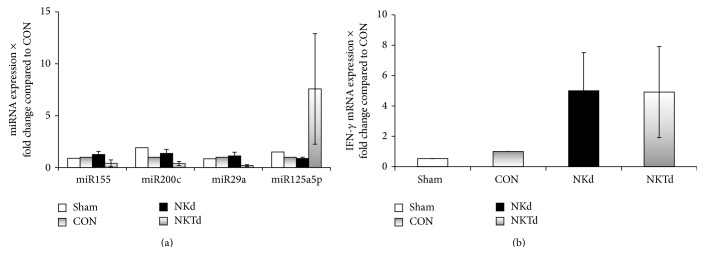
Expression of miRNA and of mRNA of IFN-*γ* in splenocytes. Splenocyte total RNA was extracted. (a) Τhe expression levels of miR-155, miR-200ca, miR-29a, and miR-125a-5p were quantified by TaqMan miRNA assays. Data was normalized to the endogenous control sno202 and fold changes of miRNA expression relative to the CON group were calculated by the ΔΔCT method. (b) Expression of* IFN-γ* mRNA in splenocytes. Splenocyte total RNA was extracted and the expression level of* IFN-γ* was quantified by TaqMan assays. Data was normalized to the endogenous control GAPDH and fold changes of mRNA expression relative to the CON group were calculated by the ΔΔCT method. Mice were pretreated with nonspecific IgG (CON, *n* = 1); with anti-asialo GM1 antibody (NKd, *n* = 2); or with anti-CD1d antibody (NKTd, *n* = 2). Results are compared with sham-operated mice (Sham, *n* = 1).

To further investigate the mechanism that may regulate the increased IFN-*γ* production observed upon blockade of CD1d activation of NKT cells during sepsis, we compared the expression of* IFN-γ* mRNA and of specific immune-related miRNAs in splenocytes between samples from the four different groups. Interestingly, miR-155, miR-200c, and miR-29a were found to be downregulated in the splenocytes of the NKTd group, by 60% to 80% compared to controls (Figure 4(a)). In contrast, miR-125a-5p was upregulated in NKTd splenocytes 8-fold compared to all other groups. Following quantitation of* IFN-γ* mRNA in the same samples, we found a 5-fold increase in the NKTd animals (Figure 4(b)), which correlates negatively to miR-155, miR-200c, and miR-29a downregulation and positively to miR-125a-5p induction. These data provide an indication that inhibition of CD1d-dependent NKT cell activation may be associated with specific miRNA alterations during sepsis, which may be implicated in the transcriptional regulation of IFN-*γ* production.

## 4. Discussion

The presented results clearly show a major role of the NK/NKT cell balance in the outcome of experimental pneumococcal pneumonia. Depletion of NK cells protected animals from death; however, this did not occur with animals where NKT cell activation was blocked. Differences in survival were related to an effect on* S. pneumoniae* tissue burden and on IFN-*γ* production.

The findings of our study demonstrate a beneficial response of NK cell depletion in a nonimmunocompromised experimental model of pneumococcal pneumonia. However, a similar protective effect from NK cell depletion has been shown in experimental sepsis induced after cecal ligation and puncture (CLP) or after challenge with LPS or* Ehrlichia* spp. [20–27]. In these studies, depletion of NK cells was accompanied by attenuated inflammatory responses.

Also, our results pinpoint a role of the NK/NKT cell balance on tissue bacterial outgrowth. More precisely, depletion of NK cells was accompanied by decreased bacterial growth and blockade of NKT cell activation primed bacterial growth. Godshall et al. [16] showed that, after* in vivo* NK cell depletion using the anti-asialo GM1 antibody, bacterial loads in the liver and peritoneal fluid were increased 4 hours after CLP, compared with the control group. However, 8 and 18 hours after CLP bacterial loads were similar between the two groups and the same was true for mortality. It has been suggested that a complex interaction between NK cells and macrophages may promote bacterial clearance [28]. However, in our study, despite a higher bacterial burden in the NKTd group, we did not find a statistically significant survival disadvantage in this group. It is possible that, apart from a higher systemic bacterial burden, alterations in innate immune response mechanisms mediated by blockade of CD1d-dependent activation of NKT cells may play a role in the outcome of pneumococcal pneumonia and sepsis.

The importance of NK cells in the clinical setting of CAP has recently been confirmed in a large Greek cohort of 505 patients; patients underwent analysis of circulating cell subpopulations by flow cytometry within the first 24 hours from the diagnosis of sepsis and results differed according to the type of underlying infection. Analysis indicated a significant decrease of the circulating number of NK cells in patients with CAP upon worsening from sepsis to severe sepsis/shock. This was a unique feature of CAP not found in sepsis due to other infectious causes [29].

Existing evidence coming from animal studies has provided contradictory results for the role of NKT cells. It is reported that CD1d blockade prior to sepsis had a positive impact on survival in experimental CLP, which correlated with a decrease in spleen NKT cells and in circulating IL-6 and IL-10 [30, 31]. On the other hand, Kawakami et al. [32] investigated the role of NKT cells in* S. pneumoniae* infection using J*α*281^−/−^ mice (deficient in Valpha14+ NKT cells) and found that these animals were significantly more susceptible to infection when compared with controls, possibly due to decreased recruitment of neutrophils. In our study, the animals that received the anti-CD1d antibody were not protected from infection. Inhibition of CD1d-dependent recognition of* S. pneumoniae* glycolipids by NKT cells may be associated with the decreased survival in the anti-CD1d treated animals [8].

Following stimulation of V*α*14*i*NKT cells with aGalCer* in vivo*, large amounts of IFN-*γ* are produced from NK cells [33–36]. Also, splenocyte stimulation with aGalCer and TLR agonists induced IFN-*γ* production in CD8+ T cells and NK cells [37]. However, the interaction between NK and NKT cells after physiologic activation of the latter in the absence of aGalCer, which is a potent TCR agonist, is less well studied. It is possible that the type of TCR signal can affect the profile of cytokines produced by NKT cells, favoring either Th1 or Th2 responses [36, 38–40]. It has been shown in two studies that staphylococcal and streptococcal superantigens can activate mouse and human iNKT cells in a CD1d-independent manner via MHC II/TCR interaction. This CD1d-independent activation of iNKT cells by bacterial superantigens resulted in a Th1-type response with increased IFN-*γ* production and acute lung injury in mice [41, 42]. In our study, blockade of CD1d-dependent activation of NKT cells resulted in an increase in spleen NK cells and was associated with a higher IFN-*γ* production in both the serum and splenocyte supernatants. An exaggerated Th1 response, as evidenced by the increase of NK cells and IFN-*γ* production, upon blockade of CD1d-dependent NKT cell activation could potentially explain the survival disadvantage in this group. Our findings corroborate the results of a recent study of 103 patients with sepsis. NK cells were isolated and* ex vivo* stimulated for cytokine production; patients who were overproducing IFN-*γ* had an almost 3-fold greater risk of dying [43]. However, other investigators have shown that NKT cells may not contribute to acute inflammation induced by Gram-negative bacteria [21, 23]. It has been hypothesized that the role of NK cells in bacterial infection and sepsis may differ according to the type of pathogen or the site of infection [29, 44].

On the molecular level, the expression of* IFN-γ* mRNA was induced in the spleen of animals where CD1d activation of NKT cells was blocked. A similar trend was also noted in the anti-asialo GM1 treated group. Furthermore, we studied whether miRNAs were involved in the regulation of* IFN-γ* production. We chose to study specific miRNAs involved in IFN-*γ* production or relevant to NK and NKT cells. Our study revealed that both spleen miR-200c and miR-29a were downregulated upon inhibition of NKT cell activation, which correlated with enhanced IFN-*γ* production both in serum and by splenocytes. This evidence is in agreement with the role of miR-200c and miR-29a in bacterial infection, where it has been associated with decreased IFN-*γ* production by NK cells in two independent studies [45, 46]. A caveat of our study is that because of the limited number of samples molecularly tested, our results can only provide an impetus for further research on the role of miRNAs in the regulation of IFN-*γ* production during blockade of CD1d activation.

Another limitation of the study is that treatment with anti-asialo GM1 might also affect other types of cells that express asialo GM1, like a small subset of CD8+ T cells, *γδ* T cells, or basophils [47]. However, treatment with anti-asialo GM1 did not seem to influence the number of IFN-*γ* secreting CD8+ T cells in the lung [48].

## 5. Conclusion

The presented findings provide novel insights into the mechanisms of regulation of IFN-*γ* production and the contributions of NK and NKT cells in experimental pneumococcal pneumonia. Greater IFN-*γ* production occurs upon pneumococcal challenge in mice following blockade of CD1d-dependent NKT cell activation. Changes in NK cell expression of specific miRNAs are likely responsible for the upregulation of* IFN-γ* mRNA and protein levels. These findings help expand our current knowledge regarding the role of NK and NKT cells in the host response against invasive, extracellular bacterial pathogens such as* Streptococcus pneumoniae*. Clarifying the role of these cell populations in severe infections could offer new therapeutic targets for the treatment of severe CAP.

## Abbreviations

cfu: Colony forming units
EDTA: Ethylenediaminetetraacetic acid
IFN-*γ*: Interferon-gamma
IL: Interleukin
LPS: Lpopolysaccharide
miR: MicroRNA
NK: Natural killer
PBS: Phosphate buffered saline
PE: Phycoerythrin
PI: Propidium iodide
TNF-*α*: Tumour necrosis factor-alpha.

## References

[1] Angus D. C., Linde-Zwirble W. T., Lidicker J., Clermont G., Carcillo J., Pinsky M. R. (2001). Epidemiology of severe sepsis in the United States: analysis of incidence, outcome, and associated costs of care. *Critical Care Medicine*.

[2] Martin G. S., Mannino D. M., Eaton S., Moss M. (2003). The epidemiology of sepsis in the United States from 1979 through 2000. *The New England Journal of Medicine*.

[3] Degli-Esposti M. A., Smyth M. J. (2005). Close encounters of different kinds: dendritic cells and NK cells take centre stage. *Nature Reviews Immunology*.

[4] Moretta A., Marcenaro E., Parolini S., Ferlazzo G., Moretta L. (2008). NK cells at the interface between innate and adaptive immunity. *Cell Death and Differentiation*.

[5] Mattner J., DeBord K. L., Ismail N. (2005). Exogenous and endogenous glycolipid antigens activate NKT cells during microbial infections. *Nature*.

[6] Leung B., Harris H. W. (2010). NKT cells in sepsis. *Clinical and Developmental Immunology*.

[7] Godfrey D. I., Hammond K. J. L., Poulton L. D., Smyth M. J., Baxter A. G. (2000). NKT cells: facts, functions and fallacies. *Immunology Today*.

[8] Kinjo Y., Illarionov P., Vela J. L. (2011). Invariant natural killer T cells recognize glycolipids from pathogenic Gram-positive bacteria. *Nature Immunology*.

[9] Wesley J. D., Robbins S. H., Sidobre S., Kronenberg M., Terrizzi S., Brossay L. (2005). Cutting edge: IFN-*γ* signaling to macrophages is required for optimal V*α*14i NK T/NK cell cross-talk. *The Journal of Immunology*.

[10] Schnitger A. K. D., Machova A., Mueller R. U. (2011). Listeria monocytogenes infection in macrophages induces vacuolar-dependent host miRNA response. *PLoS ONE*.

[11] Sullivan R. P., Leong J. W., Fehniger T. A. (2013). MicroRNA regulation of natural killer cells. *Frontiers in Immunology*.

[12] Zheng Q., Zhou L., Mi Q. S. (2012). MicroRNA miR-150 is involved in Valpha14 invariant NKT cell development and function. *Journal of Immunology*.

[13] Trotta R., Chen L., Ciarlariello D. (2012). miR-155 regulates IFN-*γ* production in natural killer cells. *Blood*.

[14] Mohler J., Azoulay-Dupuis E., Amory-Rivier C. (2003). *Streptococcus pneumoniae* strain-dependent lung inflammatory responses in a murine model of pneumococcal pneumonia. *Intensive Care Medicine*.

[15] Kerr A. R., Kirkham L. A. S., Kadioglu A. (2005). Identification of a detrimental role for NK cells in pneumococcal pneumonia and sepsis in immunocompromised hosts. *Microbes and Infection*.

[16] Godshall C. J., Scott M. J., Burch P. T., Peyton J. C., Cheadle W. G. (2003). Natural killer cells participate in bacterial clearance during septic peritonitis through interactions with macrophages. *Shock*.

[17] Nieuwenhuis E. E. S., Matsumoto T., Exley M. (2002). CD1d-dependent macrophage-mediated clearance of *Pseudomonas aeruginosa* from lung. *Nature Medicine*.

[18] Livak K. J., Schmittgen T. D. (2001). Analysis of relative gene expression data using real-time quantitative PCR and the 2-ΔΔCT method. *Methods*.

[19] Jeong D. G., Jeong E. S., Seo J. H., Heo S. H., Choi Y. K. (2011). Difference in resistance to *Streptococcus pneumoniae* infection in mice. *Laboratory Animal Research*.

[20] Chiche L., Forel J.-M., Thomas G. (2011). The role of natural killer cells in sepsis. *Journal of Biomedicine and Biotechnology*.

[21] Etogo A. O., Nunez J., Lin C. Y., Toliver-Kinsky T. E., Sherwood E. R. (2008). NK but not CD1-restricted NKT cells facilitate systemic inflammation during polymicrobial intra-abdominal sepsis. *The Journal of Immunology*.

[22] Heremans H., Dillen C., Van Damme J., Billiau A. (1994). Essential role for natural killer cells in the lethal lipopolysaccharide-induced Shwartzman-like reaction in mice. *European Journal of Immunology*.

[23] Emoto M., Miyamoto M., Yoshizawa I. (2002). Critical role of NK cells rather than V*α*14^+^NKT cells in lipopolysaccharide-induced lethal shock in mice. *Journal of Immunology*.

[24] Enoh V. T., Fairchild C. D., Lin C. Y., Varma T. K., Sherwood E. R. (2006). Differential effect of imipenem treatment on wild-type and NK cell-deficient CD8 knockout mice during acute intra-abdominal injury. *The American Journal of Physiology—Regulatory Integrative and Comparative Physiology*.

[25] Sherwood E. R., Enoh V. T., Murphey E. D., Lin C. Y. (2004). Mice depleted of CD8^+^ T and NK cells are resistant to injury caused by cecal ligation and puncture. *Laboratory Investigation*.

[26] Carson W. E., Yu H., Dierksheide J. (1999). A fatal cytokine-induced systemic inflammatory response reveals a critical role for NK cells. *The Journal of Immunology*.

[27] Stevenson H. L., Estes M. D., Thirumalapura N. R., Walker D. H., Ismail N. (2010). Natural killer cells promote tissue injury and systemic inflammatory responses during fatal *Ehrlichia*-induced toxic shock-like syndrome. *The American Journal of Pathology*.

[28] Scott M. J., Hoth J. J., Gardner S. A., Peyton J. C., Cheadle W. G. (2003). Natural killer cell activation primes macrophages to clear bacterial infection. *The American Surgeon*.

[29] Gogos C., Kotsaki A., Pelekanou A. (2010). Early alterations of the innate and adaptive immune statuses in sepsis according to the type of underlying infection. *Critical Care*.

[30] Rhee R. J., Carlton S., Lomas J. L. (2003). Inhibition of CD1d activation suppresses septic mortality: a role for NK-T cells in septic immune dysfunction. *Journal of Surgical Research*.

[31] Hu C. K., Venet F., Heffernan D. S. (2009). The role of hepatic invariant NKT cells in systemic/local inflammation and mortality during polymicrobial septic shock. *Journal of Immunology*.

[32] Kawakami K., Yamamoto N., Kinjo Y. (2003). Critical role of V*α*14^+^ natural killer T cells in the innate phase of host protection against *Streptococcus pneumoniae* infection. *European Journal of Immunology*.

[33] Carnaud C., Lee D., Donnars O. (1999). Cutting edge: cross-talk between cells of the innate immune system: NKT cells rapidly activate NK cells. *The Journal of Immunology*.

[34] Eberl G., MacDonald H. R. (2000). Selective induction of NK cell proliferation and cytotoxicity by activated NKT cells. *European Journal of Immunology*.

[35] Matsuda J. L., Gapin L., Baron J. L. (2003). Mouse V*α*14*i* natural killer T cells are resistant to cytokine polarization *in vivo*. *Proceedings of the National Academy of Sciences of the United States of America*.

[36] Kronenberg M. (2005). Toward an understanding of NKT cell biology: progress and paradoxes. *Annual Review of Immunology*.

[37] Ando T., Ito H., Ohtaki H., Seishima M. (2013). Toll-like receptor agonists and alpha-galactosylceramide synergistically enhance the production of interferon-gamma in murine splenocytes. *Scientific Reports*.

[38] Stanic A. K., Shashidharamurthy R., Bezbradica J. S. (2003). Another view of T cell antigen recognition: cooperative engagement of glycolipid antigens by Va14Ja18 natural T(iNKT) cell receptor [corrected]. *Journal of Immunology*.

[39] Schmieg J., Yang G., Franck R. W., Tsuji M. (2003). Superior protection against malaria and melanoma metastases by a C-glycoside analogue of the natural killer T cell ligand alpha-Galactosylceramide. *The Journal of Experimental Medicine*.

[40] Miyamoto K., Miyake S., Yamamura T. (2001). A synthetic glycolipid prevents autoimmune encephalomyelitis by inducing T_H_2 bias of natural killer T cells. *Nature*.

[41] Hayworth J. L., Mazzuca D. M., Vareki S. M., Welch I., McCormick J. K., Haeryfar S. M. (2012). CD1d-independent activation of mouse and human iNKT cells by bacterial superantigens. *Immunology and Cell Biology*.

[42] Rieder S. A., Nagarkatti P., Nagarkatti M. (2011). CD1d-independent activation of invariant natural killer T cells by Staphylococcal enterotoxin B through major histocompatibility complex class II/T cell receptor interaction results in acute lung injury. *Infection and Immunity*.

[43] Giannikopoulos G., Antonopoulou A., Kalpakou G. (2013). The functional role of natural killer cells early in clinical sepsis. *Acta Pathologica, Microbiologica et Immunologica Scandinavica*.

[44] Holub M., Klučková Z., Helcl M., Přihodov J., Rokyta R., Beran O. (2003). Lymphocyte subset numbers depend on the bacterial origin of sepsis. *Clinical Microbiology and Infection*.

[45] Huang Y., Lei Y., Zhang H., Hou L., Zhang M., Dayton A. I. (2011). MicroRNA regulation of STAT4 protein expression: rapid and sensitive modulation of IL-12 signaling in human natural killer cells. *Blood*.

[46] Ma F., Xu S., Liu X. (2011). The microRNA miR-29 controls innate and adaptive immune responses to intracellular bacterial infection by targeting interferon-gamma. *Nature Immunology*.

[47] Nishikado H., Mukai K., Kawano Y., Minegishi Y., Karasuyama H. (2011). NK cell-depleting anti-asialo GM1 antibody exhibits a lethal off-target effect on basophils in vivo. *The Journal of Immunology*.

[48] Byrne P., McGuirk P., Todryk S., Mills K. H. G. (2004). Depletion of NK cells results in disseminating lethal infection with *Bordetella pertussis* associated with a reduction of antigen-specific Th1 and enhancement of Th2, but not Tr1 cells. *European Journal of Immunology*.

